# Pod-based e-liquids impair human vascular endothelial cell function

**DOI:** 10.1371/journal.pone.0280674

**Published:** 2023-01-26

**Authors:** Sana Majid, Robert M. Weisbrod, Jessica L. Fetterman, Rachel J. Keith, Syed H. M. Rizvi, Yuxiang Zhou, Leili Behrooz, Rose Marie Robertson, Aruni Bhatnagar, Daniel J. Conklin, Naomi M. Hamburg

**Affiliations:** 1 Evans Department of Medicine and Whitaker Cardiovascular Institute, Boston University School of Medicine, Boston, MA, United States of America; 2 University of Louisville School of Medicine, Louisville, KY, United States of America; 3 American Heart Association, Dallas, TX, United States of America; University of Concepcion Faculty of Medicine: Universidad de Concepcion Facultad de Medicina, CHILE

## Abstract

Pod-based electronic (e-) cigarettes more efficiently deliver nicotine using a protonated formulation. The cardiovascular effects associated with these devices are poorly understood. We evaluated whether pod-based e-liquids and their individual components impair endothelial cell function. We isolated endothelial cells from people who are pod users (n = 10), tobacco never users (n = 7), and combustible cigarette users (n = 6). After a structured use, pod users had lower acetylcholine-mediated endothelial nitric oxide synthase (eNOS) activation compared with never users and was similar to levels from combustible cigarette users (overall P = 0.008, P = 0.01 pod vs never; P = 0.96 pod vs combustible cigarette). The effects of pod-based e-cigarettes and their constituents on vascular cell function were further studied in commercially available human aortic endothelial cells (HAECs) incubated with flavored JUUL e-liquids or propylene glycol (PG):vegetable glycerol (VG) at 30:70 ratio with or without 60 mg/mL nicotine salt for 90 min. A progressive increase in cell death with JUUL e-liquid exposure was observed across 0.0001–1% dilutions; PG:VG vehicle with and without nicotine salt induced cell death. A23187-stimulated nitric oxide production was decreased with all JUUL e-liquid flavors, PG:VG and nicotine salt exposures. Aerosols generated by JUUL e-liquid heating similarly decreased stimulated nitric oxide production. Only mint flavored e-liquids increased inflammation and menthol flavored e-liquids enhanced oxidative stress in HAECs. In conclusion, pod e-liquids and their individual components appear to impair endothelial cell function. These findings indicate the potential harm of pod-based devices on endothelial cell function and thus may be relevant to cardiovascular injury in pod type e-cigarette users.

## Introduction

Pod-based electronic (e-) cigarettes such as JUUL are comprised of heat resistant disposable cartridges that contain heating coils and utilize nicotine salts rather than the conventional freebase nicotine [1]. These devices, similar to prior generations of e-cigarettes, contain propylene glycol (PG), vegetable glycerol (VG) and flavoring chemicals. Many of these constituents are harmful or potentially harmful and toxicities associated with e-liquid constituents, including PG:VG and flavorings have been reported [2–5]. The differential composition of flavoring chemicals and the ratio of PG:VG used in e-liquids may contribute varyingly to cellular harm [6, 7].

The cardiovascular system is subject to toxicant exposure through the circulating bloodstream. Alterations in endothelial function induced by circulating toxins are important mediators of cardiovascular health [8]. Endothelial dysfunction contributes to atherogenesis and precedes cardiovascular events, and is characterized by an inflammatory response, oxidative stress, and decreased nitric oxide (NO) production [9, 10]. Combustible cigarette consumption has been shown to impair endothelial function [11, 12]. Emerging evidence links e-cigarette use to early markers of vascular harm, including endothelial dysfunction [13–16]. The effects of pod-based e-cigarettes and their corresponding nicotine salts on the vascular endothelium remains largely unknown.

In our study, we evaluated the effects of pod-based e-liquids and their constituents on vascular endothelial cells. We assessed endothelial NO synthase (eNOS) activation in freshly isolated venous endothelial cells from human participants who were pod users. Additionally, we tested endothelial cytotoxicity by measures of cellular viability, oxidative stress, inflammation, and nitric oxide (NO) production upon e-liquid exposure. We find that pod use impairs eNOS activation, and exposure of cultured cells to pod based e-liquids and their individual components alters parameters indicative of endothelial cell dysfunction.

## Methods

### Study participants and tobacco product exposure

We enrolled human participants between the ages of 21 and 45 who were without cardiovascular disease or risk. Participants were categorized as never users of tobacco products, pod-based e-cigarette users, or combustible cigarette users. Pod-based e-cigarette users must have been using product for at least 6 months for at least 5 days per week. Combustible cigarette users must have been smoking for at least 6 months at least 5 days per week without concurrent usage of e-cigarettes for at least 6 months.

To evaluate tobacco product use and eNOS activation, participants were instructed to use their own product that they brought to the study visit in a structured fashion for 10 minutes. Pod-based e-cigarette users (both dual and exclusive users) were instructed to bring the type of pod they used most frequently. Users of combustible cigarettes smoked their usual cigarette brand. Controls followed a similar timing schedule of testing but were not exposed to any tobacco product and puffed on a straw. The study did not constitute a clinical trial as individuals used their own products.

### Ethics statement

All participants gave written informed consent as approved by the University of Louisville and Boston University Medical Campus Institutional Review Boards, and all research was conducted according to the principles upheld by the Declaration of Helsinki.

### Venous endothelial cell biopsy

As previously described [17–19], venous endothelial cells were freshly isolated from the study participants following product use. Briefly, a 0.018-inch J-wire (Arrow International, Reading, PA) was inserted through a 20-gauge catheter into a superficial vein of the forearm using sterile technique and was used to gently scrape the inside of the vessel. Upon removal, the J-wires are rinsed in dissociation and red blood cell lysis buffer. Microbead magnetic column separation with CD144 (Mitenyi) was used to select endothelial cells that were then applied to poly-L-lysine coated slides (Sigma, St. Louis, MO), and either stimulated or unstimulated with 1 μM Acetylcholine (Sigma-Aldrich) for 15 min at 37°C before fixation in 4% paraformaldehyde.

### Measurement of eNOS activation by ser-1177 phosphorylation

Fixed slides were thawed and rehydrated with 50 mM glycine (Sigma-Aldrich) in PBS for 10 min. The cells were permeabilized with 0.1% Triton X-100, washed with 50mM glycine three times, and blocked with 0.5% BSA. The slides were then incubated overnight at 4°C with primary antibodies against phosphorylated eNOS at serine 1177 (1:100 dilution; Abcam) and anti-von Willebrand factor antibody (1:300 dilution; Dako). The slides were washed with 50 mM glycine and incubated at 37°C with the corresponding Alexa Fluor 488 and Alexa Fluor 594 antibodies (1:200 dilution; Invitrogen). The slides were washed again with 50 mM glycine and a glass cover slip was mounted with Vectashield containing 4’,6-diamidino-2-phenylindole for nuclear identification (Vector Laboratories). With each batch of patient-derived cells, we stained a control slide of cultured human aortic endothelial cells (HAECs), maintained in endothelial growth medium-2 Bullet Kit medium (EDM-2, Lonza) at 37°C with 5% CO_2_ and taken from a single index passage.

The quantification of immunofluorescence intensity has been described previously [19]. Briefly, images of cells were captured using a fluorescence microscope (Nikon Eclipse TE2000-E) at x20 with a Photometric CoolSnap HQ2 Camera (Photometrics) and intensity was quantified by NIS Elements AR Software (Nikon instruments) after correcting for background fluorescence. For each protein of interest, the fluorescence intensity was measured in 20 cells and averaged for each participant and condition. Intensity is expressed in arbitrary units and is the percentage of the average fluorescence intensity from the patient sample to the average fluorescence intensity of the HAEC slide stained at the same time. Data are expressed as the percentage change in eNOS fluorescence with acetylcholine stimulation. Quantification was performed while blinded to the tobacco product used by the participant (representative images in S1 Fig).

### Purchase of JUUL and nicotine salt products

We purchased the original JUUL flavors “Mango”, “Cool Mint” (referred to as Mint), “Classic Menthol” (referred to as Menthol) and “Virginia Tobacco” with 5% nicotine at vape shops in the state of Massachusetts in 2019. Nicotine salts (60 mg/mL) in 100% propylene glycol (PG) and 100% vegetable glycerol (VG) were purchased through the Liquid Nicotine Wholesalers (Phoenix, AZ) website. PG and VG were purchased from Sigma-Aldrich. To prepare a constituent mixture comparable to that reported by JUUL, Inc, we mixed 30% PG and 70% VG by volume to obtain a 30:70 PG:VG mixture. Similarly, we mixed 30% nicotine salt in 100% PG and 70% nicotine salt in 100% VG to get 60mg/mL nicotine salt in 30:70 PG:VG; henceforth, this will be referred to simply as nicotine salt.

### Tobacco product particle collection

Kentucky Reference cigarettes (3R4F and 1R5F; Tobacco Reference Products, Univ. Kentucky; Lexington, KY) and e-liquids (30:70 mixture of PG:VG; JUUL pods: Mango, Menthol, Mint, and Virginia Tobacco) were used for generating particle fractions. Mainstream cigarette smoke and e-liquid aerosols were generated using a software-controlled (FlexiWare) cigarette-smoking robot (SCI-REQ; Montreal, CAN). 3R4F and 1R5F tobacco cigarettes were humidified (24h; 60+/-1% RH; Boveda Inc., Minnetonka, MN) prior to smoking, following the International Standard of Organization (ISO) protocol (i.e., 2 s puff, 35 mL puff, 1 puff/min, 9 puffs/cigarette) [20]. E-liquids were puffed using a custom 1^st^ generation e-cigarette (Mistic bridge cartomizer, BluPlus battery, 3.7V) [21]. Each puff (3R4F: 35 mL puff; e-liquid: 91.1 mL puff; 4-sec puff) was drawn through a 10-stage Mini-MOUDI (TSI) cascade impactor (size range: 10, 5.6, 3.2, 1.8, 1.0, 0.56, 0.32, 0.18, 0.10, and 0.056 μm) at 2 lpm. The mass of particles on aluminum foil of each stage was measured gravimetrically (Mettler XPR56 ultramicrobalance; to nearest 0.1 μg; S2 Fig). Particle content on each foil was dissolved in 100% ethanol and stored at -80°C until use.

### Cell culture conditions

Commercially available (Lonza Inc, Walkersville, MD) HAECs were cultured from passages 4 to 7 in EGM-2. When cells achieved 80–90% confluence, serum was withdrawn for 4 h, and the cells were exposed to either a JUUL e-liquid, PG:VG or nicotine salt diluted in media (phenol-red-free EGM-2, Lonza) for 90 min at 37°C before measurements of apoptosis, oxidative stress, inflammation, and nitric oxide production as discussed below. Controls were HAECs in the same media conditions without e-liquid exposure. For the heated fraction studies, endothelial cells were exposed to particle fractions collected at stage 6 diluted to 0.001% for 90 minutes (stage with the highest mass concentration; 0.56 μm cutoff) as described above and stimulated NO production was evaluated.

### Evaluation of oxidative stress

HAECs were grown on 8-well slides and were exposed for 70 min to e-liquid or PG:VG vehicle prior to the addition of 5 μM MitoSOX (Invitrogen) with an additional 20 min incubation (total e-liquid exposure of 90 min). Cells were then fixed with 2% paraformaldehyde. Mean fluorescence intensity (excitation at 400 nm) of individual cells (20 cells per condition) was measured using a Nikon Eclipse TE2000 microscope. MitoSOX was measured for 10^−5^% dilution for all conditions (representative images in S3 Fig). Data are presented as the mean fluorescence intensity normalized to the mean control intensity.

### Quantification of cell death

HAECs were grown on 8-well slides, incubated with e-liquid or PG:VG vehicle for 90 minutes, fixed in 2% paraformaldehyde, and stored at -80°C. A commercially available TUNEL assay (terminal deoxynucleotidyl transferase dUTP nick-end labeling: Roche) kit was used. Cells were imaged on a fluorescence microscope (Nikon Eclipse TE2000) for fluorescein-dUTP and 4’,6-diamidino-2-phenylindole (Vector Laboratories). DNAse I (Sigma) was used as a positive control for apoptosis. A minimum of 100 cells were quantified for each condition. Data are presented as percent TUNEL-positive cells.

### Measurement of nitric oxide production

HAECs were grown on 8-well slides and after 60 min of exposure to either JUUL e-liquid, PG:VG vehicle, or particle fractions, 4,5-diaminoflourescein diacetate (1 μM; DAF-2DA, Calbiochem) was added to the cells for 30 min (totaling 90 min of toxicant exposure). After 2 washes with Hanks’ balanced salt solution (HBSS), cells were stimulated with 1 μM A23187 (Sigma) for 20 min and fixed with 2% paraformaldehyde. Mean fluorescent intensity (excitation at 500 nm) of individual cells (20 cells per condition) was measured on a fluorescence microscope (Nikon Eclipse TE2000). Data are expressed as the percentage change in DAF-2DA fluorescence with A23187 stimulation normalized to the mean of the untreated controls.

### Quantification of IL-6 activation

HAECs were grown on 6-well plates and incubated an additional 90 min in media after e-liquid or vehicle exposure, with a total of 180 min for changes in RNA expression. Cells were scraped in 350 μL RLT Plus buffer with β-mercaptoethanol, and RNA was extracted using a kit (Qiagen RNeasy Plus Micro Kit, Qiagen). RNA was eluted in 14 μL of RNase-free water and quantified with a Nanodrop spectrophotometer (ThermoFisher Scientific, average of 200 ng RNA/μL). mRNA was converted to cDNA (High-Capacity cDNA Reverse Transcription Kit, ThermoFisher Scientific) and the quantitative polymerase chain reaction was performed with the Viia7 thermal cycler (Applied Biosystems) using TaqMan Master Mix and TaqMan primers for IL-6 (ThermoFisher Scientific). The 2^-ΔΔCt^ was calculated from threshold Ct values using GAPDH as a reference gene. Data are expressed as relative quantity normalized to control conditions.

### Statistical analysis

Statistical analyses were performed using SPSS version 24.0 (IBM Corp, Armonk, NY). Data are expressed as mean ± SD, unless indicated otherwise. We evaluated each measure for normality using the Shapiro-Wilk test. For between group comparisons, variables with a normal distribution were compared using independent samples t-test or one-way ANOVA with post-hoc Tukey correction (when applicable) or *χ*^2^ testing for continuous or categorical data, respectively. For variables that were not normally distributed, Kruskal-Wallis or Mann Whitney U tests were used. Linear trends were assessed using GLM (polynomial) for parametric data and the Jonchkeere Terpsta test for nonparametric data. Adjusted analyses were performed using general linear models adjusting for age and body mass index. A two-sided *p* < 0.05 was considered statistically significant.

## Results

### Stimulated activation of eNOS function in human participants using pod e-liquids

**Fig 1:**
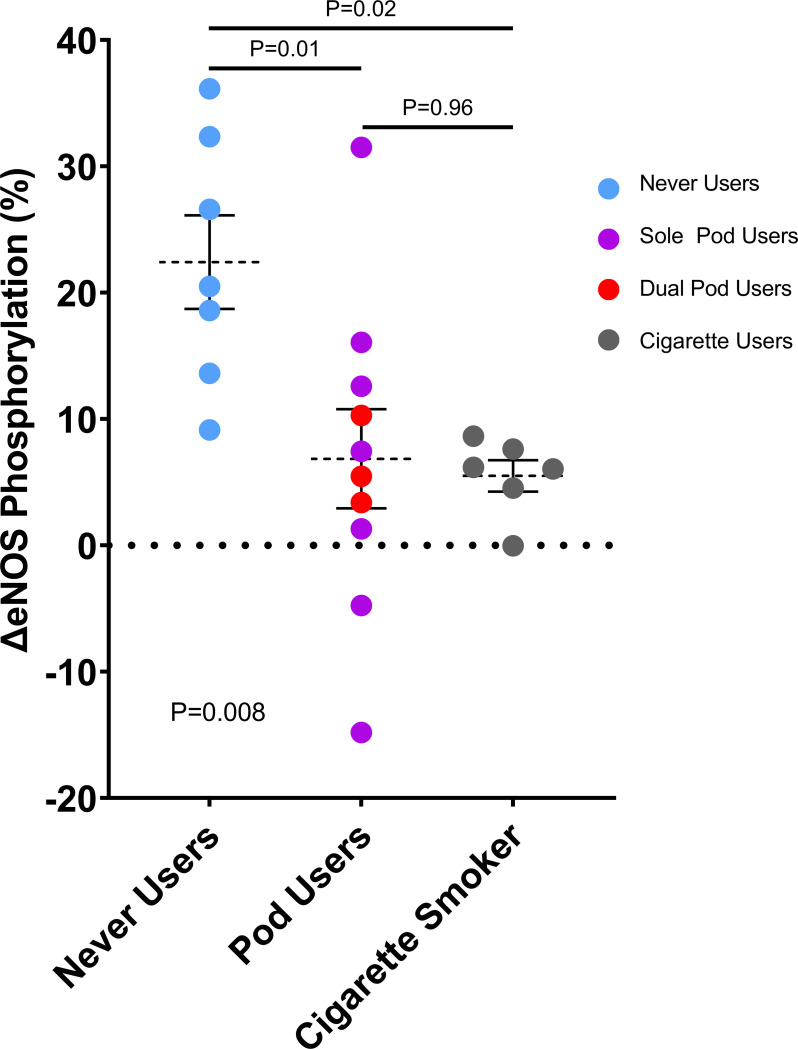
eNOS activation is impaired in endothelial cells from individuals who use pod-based e-cigarettes to a similar degree as combustible cigarette use. Ser-1177 phosphorylation was compared between the unstimulated and acetylcholine stimulated endothelial cells and evaluated as a percentage change. Data are plotted as individual measurements for each participant, mean and standard error of the mean compared by ANOVA with Tukey post-hoc testing for between group comparisons. Cigarette = combustible cigarette.

We recruited healthy human participants who were either never users of tobacco products, combustible cigarette users, or pod-based e-cigarette users as part of an ongoing observational study [22]. Pod users tended to be younger; sex and race distributions were similar across the groups (Table 1). Of the pod users, 30% were dual users of pod-based e-cigarettes and combustible cigarettes, and 70% reported using JUUL brand e-cigarettes and e-liquids. As shown in Fig 1, there was a difference in the degree of acetylcholine-induced eNOS phosphorylation at Ser-1177 in endothelial cells across the 3 groups (P = 0.008 by ANOVA). In comparison with endothelial cells from never users (22.4 ± 9.8%), stimulated eNOS phosphorylation was blunted in cells from pod-based e-cigarette users (6.8 ± 12.4%, P = 0.01) and the degree of impairment was similar to combustible cigarettes users (5.5 ± 3.0%, P = 0.96 vs pod users, P = 0.02 vs never user). The results were similar in sensitivity analyses excluding the two outliers in the pod user group. The results were similar in sensitivity analyses including only sole pod users (7.1 ± 15.1%, P = 0.04 vs never users, P = 0.96 vs combustible cigarette users). In models adjusting for age and body mass index, pod users had lower stimulated eNOS phosphorylation (adjusted mean ± SEM 8.0 ± 3.8) than never users (21.3 ± 4.3, P = 0.04) and similar to combustible cigarette users (4.9 ± 5.6, P = 0.69).

**Table 1:** Clinical characteristics.

		Never Users (n = 7)	Pod-based E-cigarette Users (n = 10)	Combustible Cigarette Users (n = 6)	P-value
**Age, y**		26 ± 2	23 ± 4	34 ± 8	0.001
**Female sex, n (%)**		5 (71)	3 (30)	2 (40)	0.2
**Race, n (%)**					0.06
	Asian	3 (43)	2 (20)	1 (17)	
	Black/African American	0 (0)	3 (30)	0 (0)	
	White	4 (57)	2 (20)	5 (83)	
	Other	0 (0)	3 (30)	0 (0)	
Body mass index, kg/m^2^		21.1 ± 2.8	23.9 ± 4.8	28.5 ± 4.7	0.02
**Dual use, n (%)**			3 (30)		
**Former combustible use, n (%)**			4 (40)		
**JUUL use, n (%)**			7 (70)		

Data expressed as mean ± SD unless indicated.

Dual use refers to pod-based e-cigarette users who reported concurrent use of combustible cigarettes on at least some days in a month on a regular basis. Non-JUUL e-cigarette brands included RELX, ZIIP, and Caviar. Five individuals who use pod-based e-cigarettes used mint/menthol, 2 used tobacco, and 3 used fruit flavored e-liquids.

### Effects on cell death across a range of pod-based e-liquid dilutions

**Fig 2:**
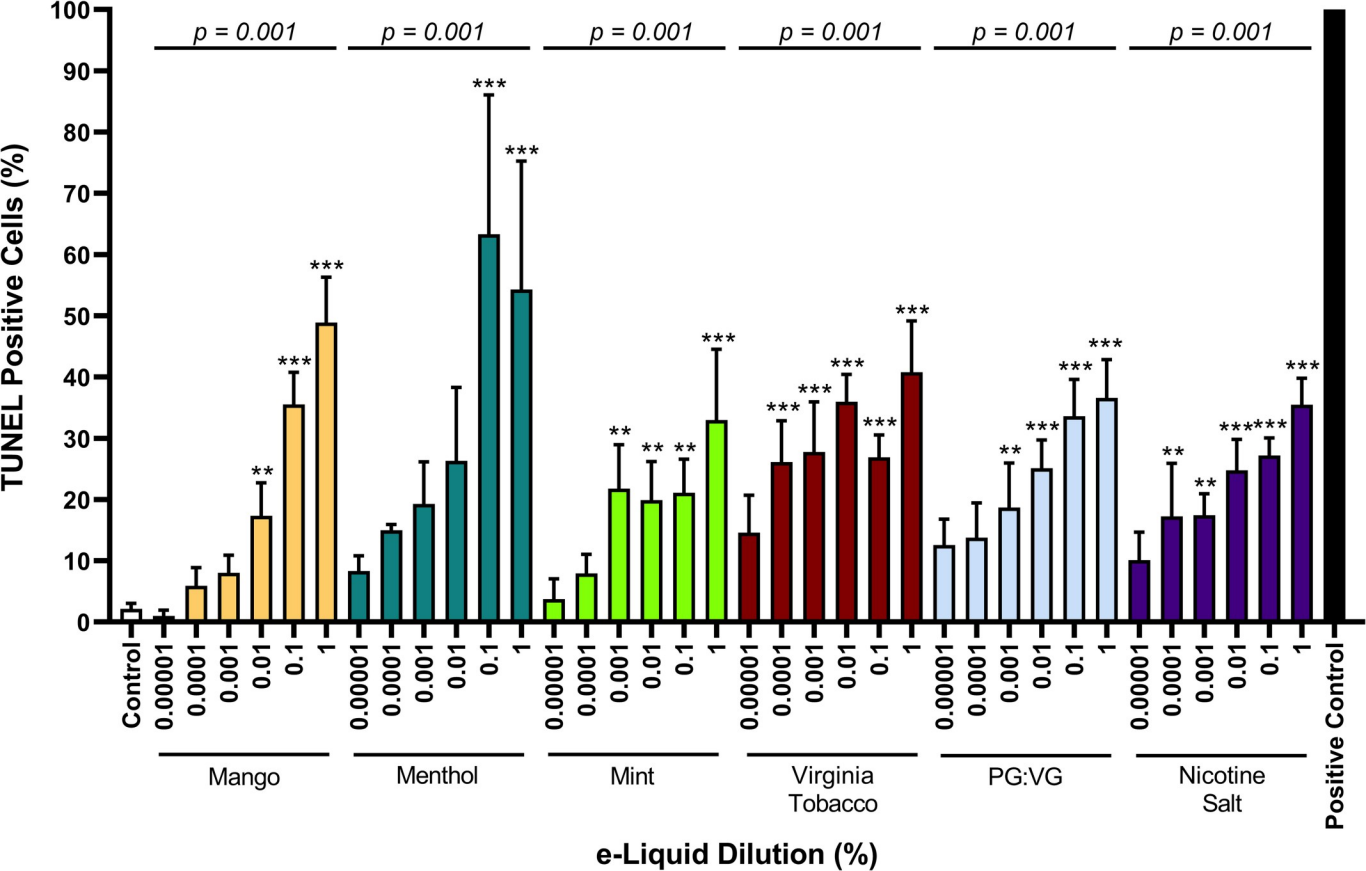
The JUUL e-liquid and its individual components induce cellular death. The percentage of cells staining positive for TUNEL (terminal deoxynucleotidyl transferase dUTP nick-end labeling) were assessed with dilutions from 1 x 10^−5^ to 1% (n = 3). DNase was used as a positive control. The percentage of apoptosis for all dilutions of each e-liquid were compared to control (* indicates p<0.05, ** indicates p<0.01, and *** indicates p<0.005). Additionally, a dose-dependent nonparametric trend test was performed for each e-liquid type (indicated by horizontal line p-value). Data are plotted as mean and SEM.

Given previous findings showing the cytotoxic effects of common tobacco product flavorings and freebase nicotine [2], we evaluated pod e-liquids with different flavors as well as the constituents of PG:VG and nicotine salt. To evaluate toxicity thresholds, we tested a range of dilutions for each e-liquid in HAECs *in vitro*. We prepared a 30% and 70% mixture of PG to VG to reflect a similar 30:60 ratio in e-liquids reported by JUUL, Inc. Nicotine salt was prepared as 60 mg/mL in 30:70 PG:VG. Though JUUL is currently available only in Classic Menthol (referred to as Menthol) and Virginia Tobacco flavors, we purchased products in 2019 and evaluated earlier JUUL e-liquids including Mango and Cool Mint and report on the findings given that fruit flavors and mint flavors remain commonly available in other pod devices. Cells were exposed to e-liquid dilutions ranging from 1 x 10^−5^% to 1%. Decreases in cell viability of at least 20% compared with unexposed controls were observed in dilutions of e-liquids as low as 1 x 10^−4^% (Fig 2). Interestingly, the PG:VG solvent alone induced a loss of cell viability to a similar extent as the JUUL e-liquids tested and nicotine salt in PG:VG, suggesting that under these conditions PG:VG vehicle alone induced cytotoxicity. Additionally, all e-liquids induced a dose-dependent increase in cell death. Due to comparable plasma nicotine levels found in individuals from previous studies [23] and limited loss of cell viability, we focused on the two lower dilutions of 1 x 10^−5^ (100,000x) and 1 x 10^−4^% (10,000x) for evaluating the effects of e-liquids on endothelial cells in the subsequent experiments.

### Effects of e-liquids on NO production in endothelial cells

**Fig 3:**
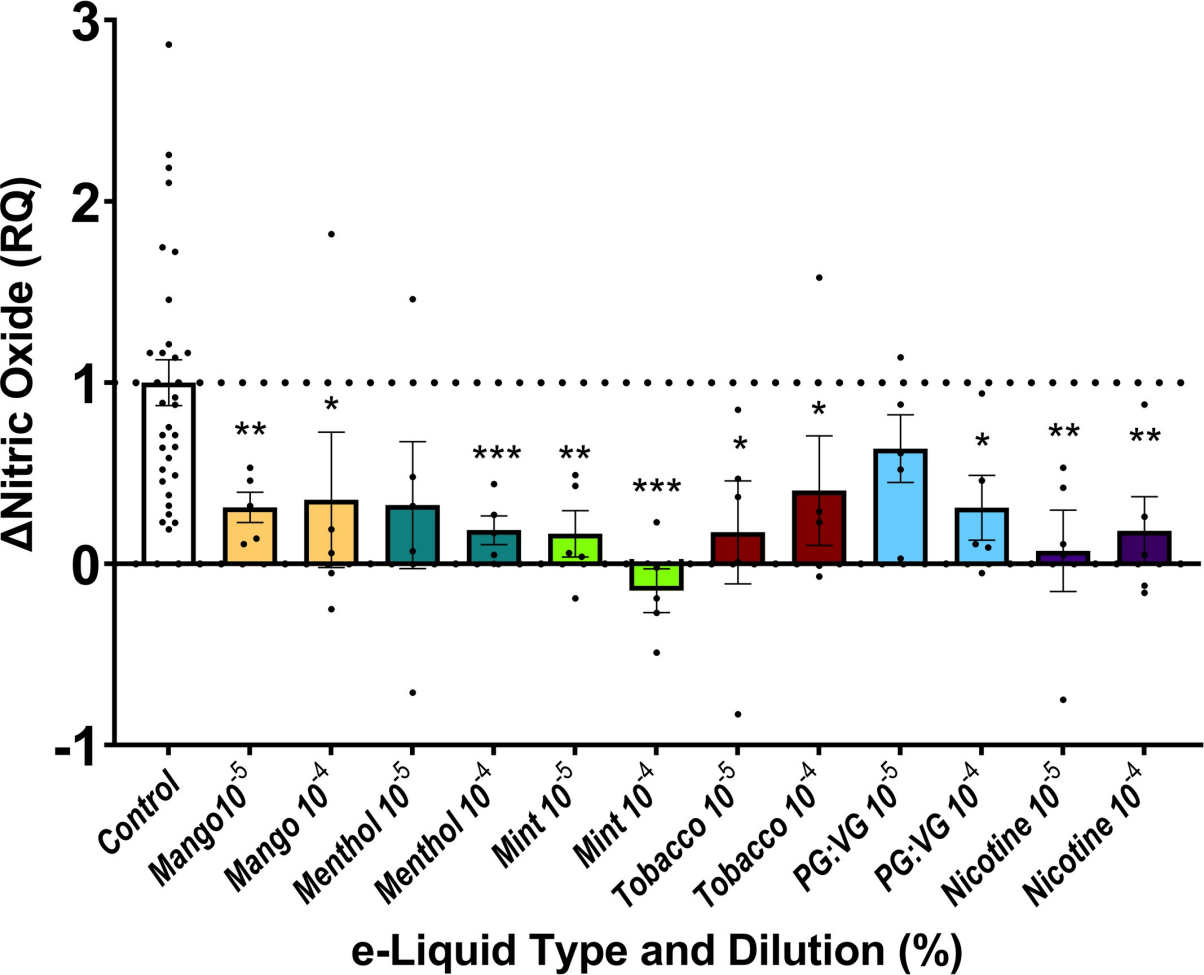
JUUL e-liquids and nicotine salt impair nitric oxide production. 4,5-diaminofluorescein diacetate fluorescence was assessed as a percentage change with A23187 stimulation. HAECs were exposed to one of the tested e-liquids at 1 x 10^−5^ and 1 x 10^−4^% dilutions (indicated as 10^−5^ and 10^−4^, respectively; n = 5), and comparisons were made between control and the tested dilutions of each e-liquid (* indicates p<0.05 and ** indicates p<0.01, *** indicates p<0.001). Data are expressed as relative change in NO production upon A23187 stimulation normalized to controls. RQ = relative quantification.

A key endothelial cell function is the ability to produce NO in response to stress or stimulation. We thus quantified NO production in response to A23187, a calcium ionophore used to stimulate eNOS in vascular endothelial cells. Relevant to control, exposure of HAECs to mango-, menthol-, mint- or tobacco-flavored JUUL e-liquids, PG:VG or nicotine salt resulted in a blunted NO response (Fig 3).

**Fig 4:**
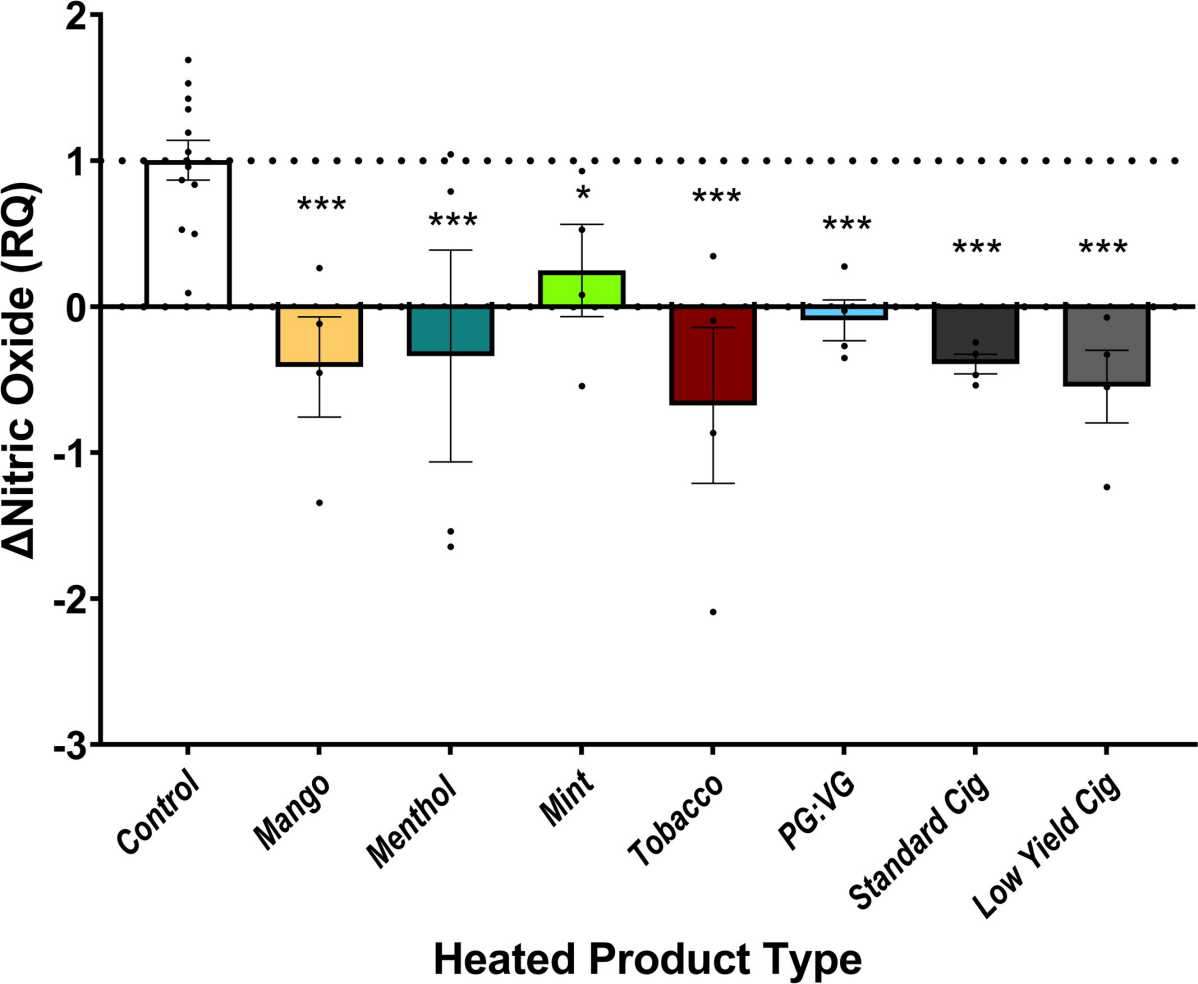
Heated particle fractions of JUUL e-liquids impair nitric oxide production. HAECs were exposed to 0.001% dilution of heated particle fractions collected at stage 6 of the 10-stage mini-MOUDI cascade impactor. 4,5-diaminoflourescein diacetate fluorescence was assessed as a percentage change with A23187 stimulation. Comparisons were made between control to each heated particle fraction (n = 4). Standard Cig = 3R4F (standard nicotine cigarette); Low Yield Cig = 1R5F (low yield nicotine cigarette). Data are plotted as mean and SEM. * indicates p<0.05, ** indicates p<0.01, and *** indicates p<0.005.

Because heating changes the chemistry of e-liquids, we examined endothelial response to particle fractions of heated and aerosolized JUUL e-liquids and PG:VG without nicotine. We prepared a 0.001% dilation of the stage 6 particle fraction, selected as the fraction with the highest mass concentration. In comparison with vehicle control, all JUUL e-liquids and PG:VG exposures impaired NO production as did the particle fractions collected from standard and low-yield nicotine reference combustible cigarettes (3R4F and 1R5F, respectively; Fig 4).

### IL-6 expression in response to e-liquid exposure

**Fig 5:**
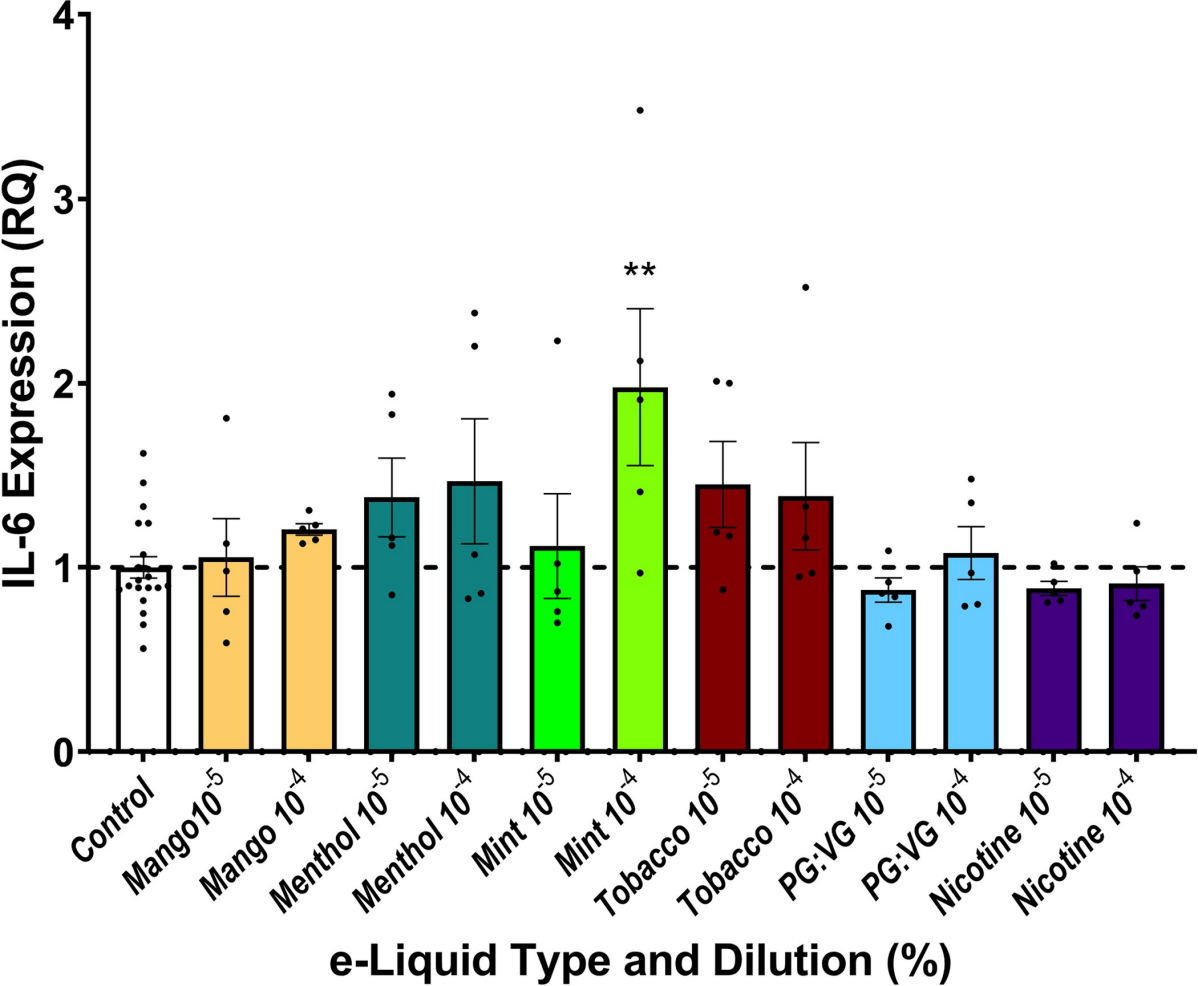
Inflammatory gene expression and pod e-liquid exposure. Interleukin-6 (IL-6) expression in endothelial cells was quantified using reverse transcription-quantitative PCR, respectively. All 2^-ΔΔCT^ values were computed relative to untreated cells, and comparisons were made between the control and tested dilutions of each e-liquid (n = 5). Data are expressed as gene expression levels normalized to matched controls. RQ = relative quantification. ** indicates p<0.01.

To examine how exposure to e-liquids affects the inflammatory response of endothelial cells, we assessed expression of IL-6, an acute phase reactant, as an indication of e-liquid induced inflammation in cultured endothelial cells. We found that compared to control, PG:VG with or without nicotine salt did not affect IL-6 expression (Fig 5). IL-6 expression was unchanged with exposure to 1 x 10^−5^% dilutions of the tested JUUL e-liquids with the exception of higher IL-6 expression at 1 x 10^−4^% dilution for mint flavored e-liquids.

### Oxidative stress in relation to e-liquid exposure

**Fig 6:**
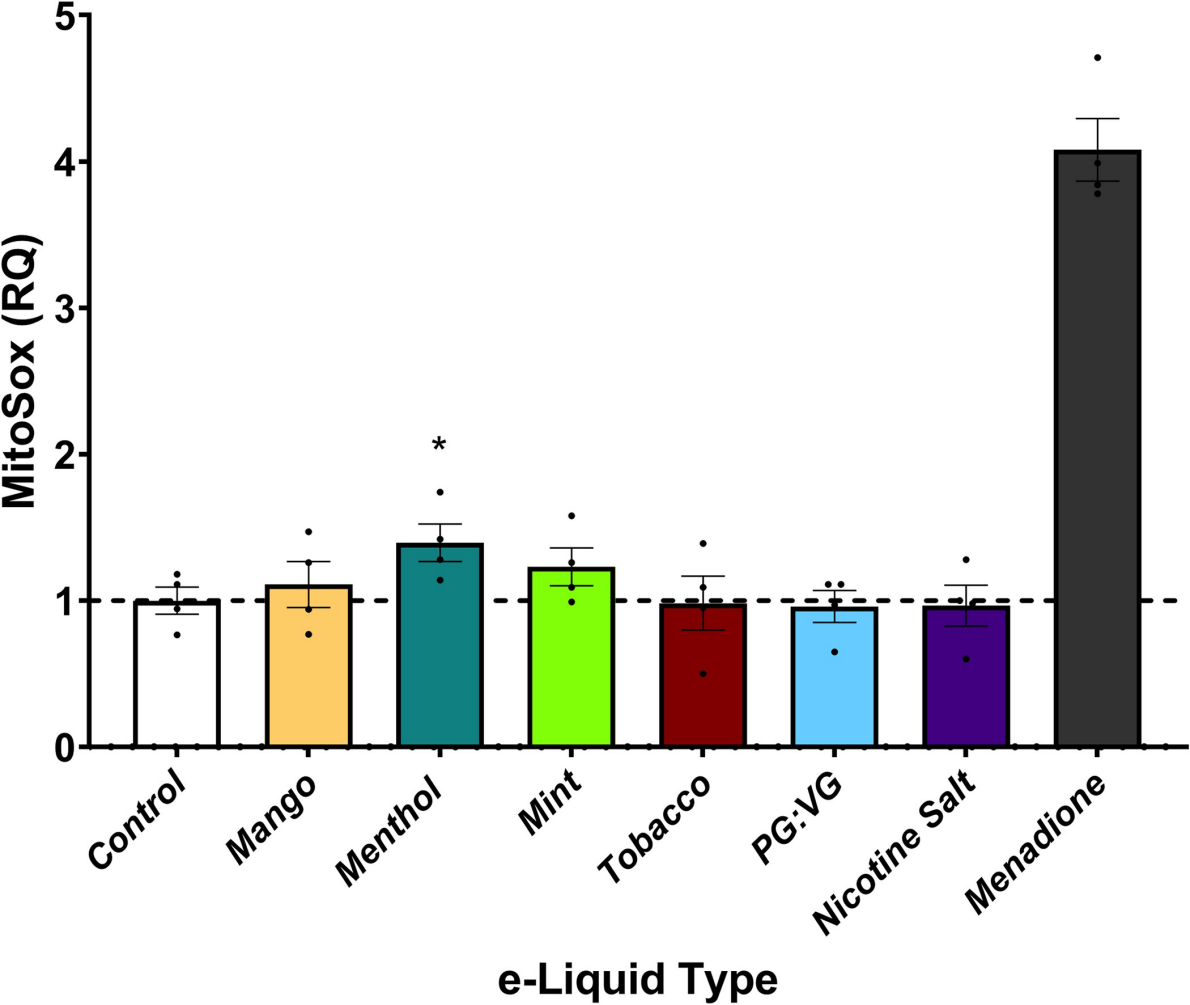
Menthol-containing JUUL e-liquids induce oxidative stress. MitoSox fluorescence was quantified in HAECs exposed to 1 x 10^−5^% dilutions of JUUL e-liquids, and the constituents PG:VG and nicotine salt (n = 4). Each e-liquid was compared to control. Data are expressed as fluorescence intensity normalized to control, dots represent individual experiments, columns and error bars are mean and SEM. * indicates p<0.05.

To examine the effects of pod-based e-liquids on a measure of oxidative stress, we evaluated mitochondrial reactive oxygen species (ROS) production using MitoSOX Red in HAECs following 90 min of e-liquid exposure. Relative to unexposed HAECs, menthol -flavored JUUL e-liquids at dilutions of 1 x 10^−5^% increased ROS production (Fig 6). In contrast, the other flavors of pod-based e-liquids and the PG:VG vehicle in the presence or absence of nicotine salt had no effect on ROS generation. Therefore, similar to e-liquid effects on inflammation, oxidative stress response may be specific to certain flavors in e-liquids.

## Discussion

In this study, we evaluated whether pod-based e-liquids impair endothelial cell function, an important prognostic marker for cardiovascular disease. Structured product use in pod users was associated with endothelial cell dysfunction in a similar fashion to combustible cigarette users. Further experiments with cultured arterial endothelial cells showed that exposure to all pod e-liquids reduced cell viability and NO production. Only selected e-liquids containing mint or menthol flavoring induced changes in inflammation and oxidative stress. These findings build upon the results of previous studies showing the effects of e-liquids on vascular endothelial cell function [16, 24, 25] by testing whole pod-based e-liquids and their heated fractions in cultured endothelial cells. Moreover, we found that the PG:VG vehicle with and without nicotine salt is not without harm. Overall, our findings suggest a dose- and flavoring-dependent impairment of endothelial cell function which may be due to diminished NO production and/or bioavailability.

Previous clinical studies evaluating the effects of e-cigarettes on vascular function in healthy smokers have demonstrated both decreased flow-mediated dilation [26, 27] and elevated pulse wave velocity, indicative of endothelial dysfunction and vascular stiffness [28]. However, it has also been reported that the use of an e-cigarette without nicotine does not impair acetylcholine-mediated vasodilation, suggesting that nicotine is a major contributor of endothelial dysfunction [29]. With pod-based e-cigarette use, one study found that JUUL was associated with a lower reactive hyperemia index and higher augmentation index, indicative of arterial stiffness in occasional smokers [30]. Although most clinical studies suggest arterial stiffness to be an earlier indicator of e-cigarette-induced harm, our study demonstrates that decreased NO production by way of decreased eNOS activation may be, in part, responsible for endothelial dysfunction at the cellular level in pod users.

*In vivo* studies have shown the overall effect of e-liquid aerosol exposure in animal models, with a relatively consistent outcome of endothelial impairment. Studies evaluating the acute and chronic effects of e-cigarettes in rodent models found increased arterial stiffness (via pulse wave velocity) [31] and reduced endothelial function (via flow-mediated dilation) [32, 33]. One acute exposure mouse study evaluating the effects of a range of e-cigarette and heated tobacco products found that all tested JUUL e-liquids and the vehicles PG and VG similarly decreased flow-mediated dilation to levels comparable to that of combustible cigarettes [34]. Similarly, a short-term exposure of female mice to aerosolized PG:VG or to formaldehyde led to aortic endothelial dysfunction *ex vivo* [21]. Acute exposure of mice to 30:70 PG:VG vehicle or JUUL e-liquid aerosols increased the urinary levels of acrolein and glycidol metabolites, suggesting that degradation of these e-cigarette constituents during aerosolization, particularly PG:VG, can lead to the formation of toxic end products [35]. Given that aldehydes, such as acrolein, are linked to the cardiovascular toxicity of cigarette smoke [35], it seems likely that changes in the composition of e-liquids during aerosolization could increase the intrinsic toxicity of e-liquids.

Both e-cigarettes and pod-mods contain e-liquids with varying levels of four main ingredients: flavorings, PG, VG, and either freebase or protonated nicotine. Previous studies measuring endothelial cell function have shown that several flavoring chemicals affect endothelial cell function, especially upon exposure to acetylpyridine, cinnamaldehyde, eugenol, or vanillin [2, 16]. The effects of nicotine, however, are conflicting; although some studies have found e-cigarette exposure induced endothelial cell impairment to be independent of nicotine content [36], others report that nicotine exposure induces cell death and triggers a pro-inflammatory response [37]. Moreover, it remains unclear whether nicotine salt alone and aerosolizing vehicles PG and VG affect vascular endothelial cells. In our study, we found that exposure to PG:VG decreased NO bioavailability with the effect being exacerbated by the addition of nicotine salt.

In the present study we demonstrate that e-liquids from commonly used pod-based products impaired endothelial cell activity and function. In previous works, e-liquids from first generation e-cigarettes were found to be cytotoxic [16, 38]. A recent study evaluating the effects of pod-based e-liquid (RELX) aerosol extracts only made comparisons to combustible cigarette smoke extract and reported that the impact of e-cigarettes was much less on the tested measures of endothelial function [39]. In contrast, another study found JUUL e-liquids to adversely affect cell viability and elevate cytoplasmic Ca^2+^ in HEK293T cells, in addition to noting differences among the flavors, with the “Mint” flavor being the most toxic [40]. Herein, compared with unexposed HAECs, we found that pod-based e-liquid exposure impaired cell viability and NO production, while exposure to mint- and menthol-flavored e-liquids also increased measures of oxidative stress and inflammation in endothelial cells, respectively. This suggests that there may be flavoring specific differences in the form of cytotoxicity.

NO is a crucial vasodilatory endothelium-derived molecule with anti-atherosclerotic and anti-aggregatory properties [41]. We found differential effects of JUUL e-liquids on IL-6 expression and oxidative stress, however, the adverse impact on cell death and NO production were similar across the different flavored JUUL e-liquids as well as the PG/VG alone. Additionally, freshly isolated endothelial cells from human participants showed reduced eNOS activation. Overall, these findings indicate reduced NO production, contrary to NO degradation, that is causing decreased NO bioavailability. This reduction in NO may be driven by NADPH oxidase, overexpression of which has been shown to inhibit acetylcholine-induced relaxation induced by eNOS activation as shown in mice exposed to e-cigarette aerosols [27]. Furthermore, eNOS activity can also be affected by limitations in tetrahydrobiopterin, an essential cofactor for proper eNOS function [42]. Additional studies are required to identify specific mechanisms by which e-liquids diminish NO production and/or bioavailability.

Our findings are also consistent with previous studies demonstrating certain flavored e-liquids to cause more harm that others [2, 16]; although the specific mechanisms by which flavors like mint or menthol exert toxicity remain unclear. These differences may be related to the production of free radicals and other reactive chemicals during their metabolism. In this regard, it was recently reported that JUUL e-cigarettes emit benzene, carbonyls and free radicals [43–46], which could lead to significant endothelial dysfunction and toxicity. The results of predictive modelling suggest the exposure to volatile organic compounds such as benzene, acrolein, and formaldehyde can affect a multitude of physiological pathways that could affect multiple health outcomes, including mental health [47]. Therefore, it may be worthwhile to evaluate the toxicant emissions of pod e-liquids and their effects on cellular function, so that appropriate product standards could be developed to limit the production of volatile organic compounds in e-cigarette aerosols.

Despite its many strengths, our study has important limitations. A majority of our work involved exposure of endothelial cells to JUUL e-liquids suspended in media though we did evaluate NO production with heated product use. We limited our testing in cell culture to JUUL e-liquids and several flavors are no longer marketed; however, flavoring compounds in fruit and mint are available in other current products including disposable pods. We used a 1^st^ generation e-cigarette to aerosolize e-liquids in order to produce heated particles fractions, the mechanics of which are different than pod-based e-cigarettes such as JUUL and may potentially impact the toxins produced. Furthermore, because we were unable to purchase nicotine salt alone rather than that prepared in solvents, we could not assess the exclusive effects of nicotine salt. Additionally, we studied only short-term exposure to e-liquid, and further studies are required to assess the long-term effects of e-liquids particularly on gene expression levels. In performing cell culture work with HAECs, we proceeded with common practices in reducing cell culture variability, it remains possible that with larger sample sizes we would have observed additional differences induced by specific e-liquids. We also did not study the differential effects of varying concentrations of nicotine salts on endothelial function. In our *ex vivo* work studying endothelial cells collected from human participants, the sample size of each tobacco use group was small, and we asked the participants to bring their own tobacco product for study purposes rather than evaluating use with a uniform e-cigarette product and e-liquid content. We did not study the baseline chronic effects in the pod-based e-cigarette users, and thereby were unable to make comparisons to baseline as a statement of acute pod e-liquid exposure. Given the small number of participants studied, we are not able to evaluate for sex-based differences. It is possible that longer duration of tobacco product use in cigarette users could influence our results; however, we observed no differences in eNOS activation in pod users compared to combustible cigarette users. Finally, the majority of the pod users used JUUL, which limits our ability to comment on other pod-based devices.

Taken together, the results of the present study provide important insights into the association of pod e-liquids composed of nicotine salts with endothelial dysfunction, with varying effects dependent on individual constituents and flavoring chemicals. Our results also suggest that endothelial impairment induced by e-cigarettes may be, in part, due to impaired eNOS activation to a degree similar to that seen in users of combustible cigarettes. These findings support regulation of tobacco products containing acidified nicotine, as well as the development of product and device standards to limit the generation of harmful or potentially harmful chemicals in e-cigarette aerosols. Additional studies are required to evaluate the long-term effects of pod usage in young adults and tobacco naïve individuals as well as to characterize particle fractions of heated e-liquids and their individual effects on endothelial function.

## Supporting information

S1 Fig Representative images of peNOS staining. In nonusers, the addition of acetylcholine clearly increases peNOS (red) fluorescence (an indicator of phosphorylation) compared to the unstimulated endothelial cell. However, this effect is not as striking among both pod users and cigarette users, as noted by the relatively similar level of fluorescence between the cells unstimulated and stimulated with acetylcholine. Blue fluorescence indicates DAPI staining for the cellular nucleus, and green fluorescence indicates von Willebrand factor for endothelial cells.
(TIF)

S2 Fig Schema of tobacco product heated particle collection. JUUL aerosol or smoke condensate is passed through the 10-stage Mini-MOUDI (TSI) cascade impactor, and particles are separated based on particle size in 10 stages. For all products, stage 6 yielded the highest mass of particles, which was dissolved in ethanol and used for testing.
(TIF)

S3 Fig Determination of oxidative stress using MitoSOX. The representative images show two replicates for control, menthol, and mint treated HAECs for 90 minutes and loaded with MitoSOX.
(TIF)
