# Supplementary figures and images for: Pod-based e-liquids impair human vascular endothelial cell function
Source: PLoS One. 2023 Jan 26;18(1):e0280674. doi: 10.1371/journal.pone.0280674 (PMC9879485; doi:10.1371/journal.pone.0280674)

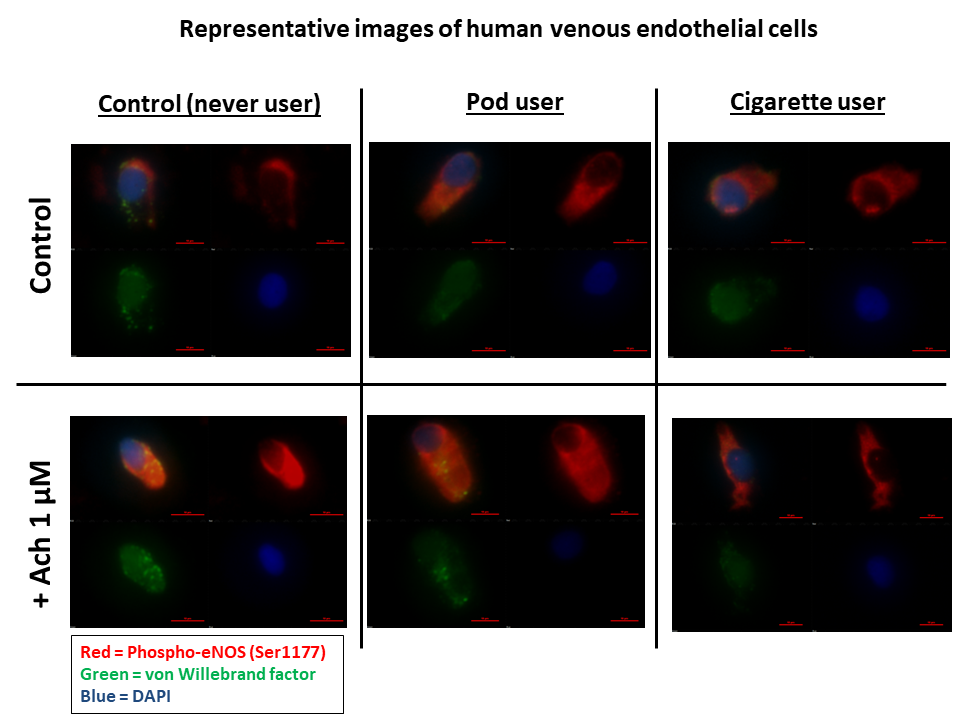

Supplement: S1 Fig — In nonusers, the addition of acetylcholine clearly increases peNOS (red) fluorescence (an indicator of phosphorylation) compared to the unstimulated endothelial cell. However, this effect is not as striking among both pod users and cigarette users, as noted by the relatively similar level of fluorescence between the cells unstimulated and stimulated with acetylcholine. Blue fluorescence indicates DAPI staining for the cellular nucleus, and green fluorescence indicates von Willebrand factor for endothelial cells. (TIF) [file pone.0280674.s001.tif]

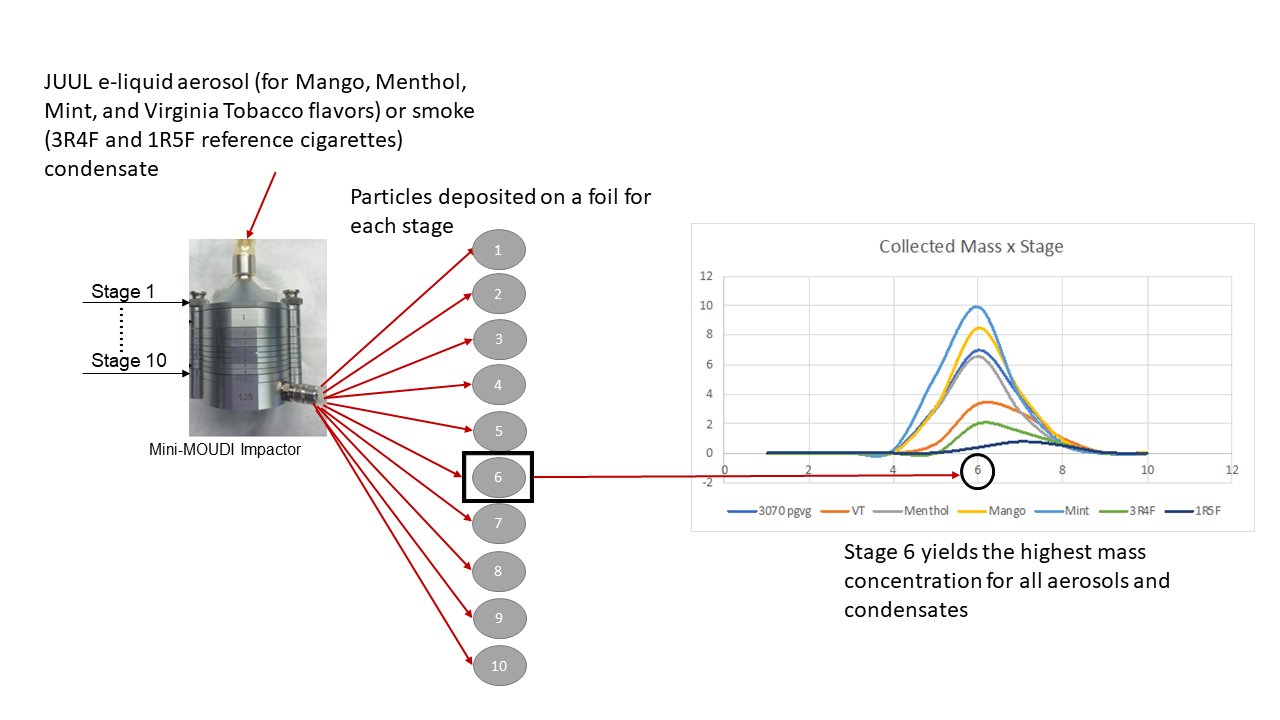

Supplement: S2 Fig — JUUL aerosol or smoke condensate is passed through the 10-stage Mini-MOUDI (TSI) cascade impactor, and particles are separated based on particle size in 10 stages. For all products, stage 6 yielded the highest mass of particles, which was dissolved in ethanol and used for testing. (TIF) [file pone.0280674.s002.tif]

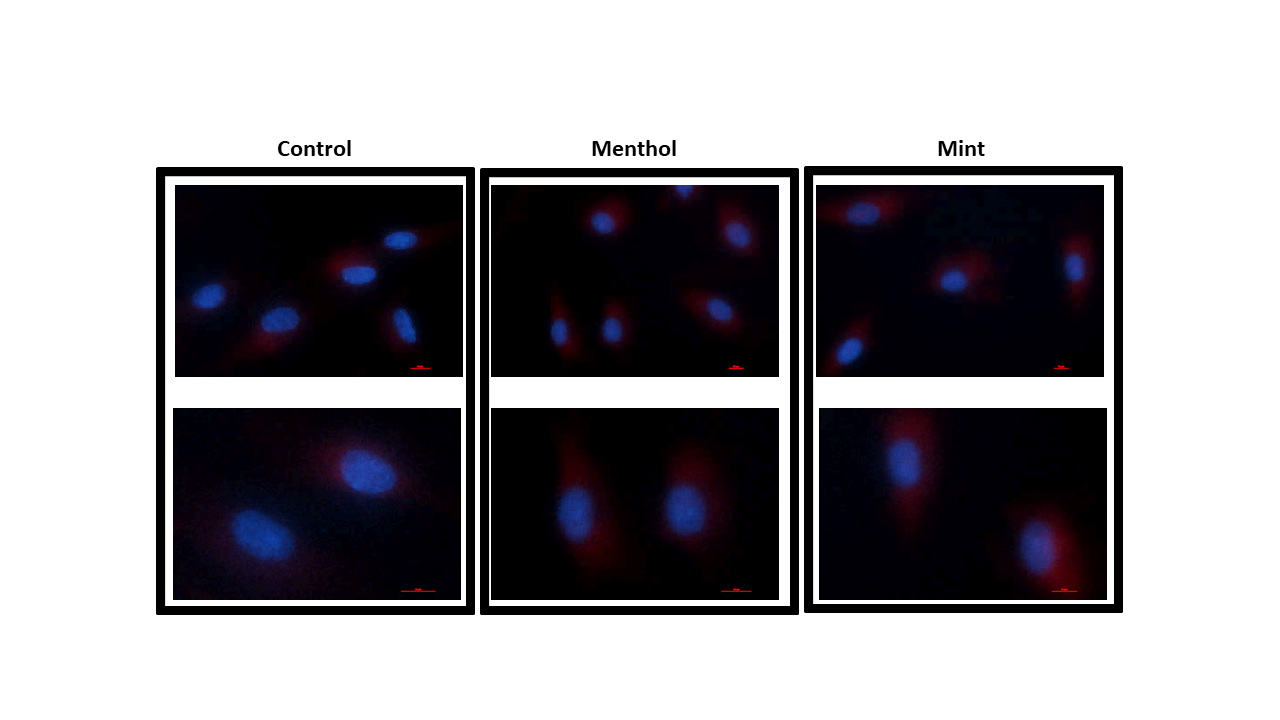

Supplement: S3 Fig — The representative images show two replicates for control, menthol, and mint treated HAECs for 90 minutes and loaded with MitoSOX. (TIF) [file pone.0280674.s003.tif]
